# A genetic switch for worker nutrition-mediated traits in honeybees

**DOI:** 10.1371/journal.pbio.3000171

**Published:** 2019-03-21

**Authors:** Annika Roth, Christina Vleurinck, Oksana Netschitailo, Vivien Bauer, Marianne Otte, Osman Kaftanoglu, Robert E. Page, Martin Beye

**Affiliations:** 1 Institute of Evolutionary Genetics, Heinrich-Heine University Dusseldorf, Düsseldorf, Germany; 2 School of Life Sciences, Arizona State University, Phoenix, Arizona, United States of America; 3 Department of Entomology and Nematology, University of California Davis, Davis, California, United States of America; New York University, UNITED STATES

## Abstract

Highly social insects are characterized by caste dimorphism, with distinct size differences of reproductive organs between fertile queens and the more or less sterile workers. An abundance of nutrition or instruction via diet-specific compounds has been proposed as explanations for the nutrition-driven queen and worker polyphenism. Here, we further explored these models in the honeybee (*Apis mellifera*) using worker nutrition rearing and a novel mutational screening approach using the clustered regularly interspaced short palindromic repeats/CRISPR-associated protein 9 (CRISPR/Cas9) method. The worker nutrition-driven size reduction of reproductive organs was restricted to the female sex, suggesting input from the sex determination pathway. Genetic screens on the sex determination genes in genetic females for size polyphenism revealed that *doublesex* (*dsx*) mutants display size-reduced reproductive organs irrespective of the sexual morphology of the organ tissue. In contrast, *feminizer* (*fem*) mutants lost the response to worker nutrition-driven size control. The first morphological worker mutants in honeybees demonstrate that the response to nutrition relies on a genetic program that is switched “ON” by the *fem* gene. Thus, the genetic instruction provided by the *fem* gene provides an entry point to genetically dissect the underlying processes that implement the size polyphenism.

## Abstract 2

In honeybees, nutrition drives dimorphic size development of reproductive organs in fertile queens and sterile workers. A study using the first induced morphological mutants in honeybees demonstrates that this developmental plasticity requires a genetic program that is switched on by the “feminizer” gene.

## Author summary

In honeybees, nutrition drives dimorphic size development of reproductive organs in fertile queens and sterile workers. The first induced morphological mutants in honeybees demonstrate that this developmental plasticity requires a genetic program that is switched “ON” by the *feminizer* (*fem*) gene.

## Introduction

Highly social insects are characterized by caste dimorphism, with morphologically and physiologically distinct reproductive queens and more or less sterile workers [1–3]. In honeybees, the development of two distinct phenotypes is controlled by different nutrition, and it is a prominent example of developmental plasticity and polyphenism [4, 5].

One major concern for the study of caste development involves explaining how a usually sterile worker and a queen that lays up to 2,000 eggs per day develop from different diet and feeding regimens [4, 6, 7]. Worker-destined larvae receive restricted amounts of a reduced sugar content diet (worker jelly [WJ]), while queen-destined larvae receive large quantities of a sugar-rich diet (royal jelly [RJ]) [8–11]. WJ and RJ drive the development of female larvae in two distinct morphs. Workers have a five-day longer developmental time, lower body mass, two small ovaries containing few ovarioles, and mid- and hind-leg structures adapted for pollen collection and transport. Queens have a five-day shorter developmental time, larger body mass, and two large ovaries that contain many more ovarioles, and they lack the pollen collection structures on the legs.

Two types of models have been proposed to explain how diets and feeding regimens mediate worker/queen development. The Nutrition/Growth model suggests that queen/worker development is driven by the amount of food and balance of nutrition [7, 11, 12], which modulate a developmental program. Queen-destined larvae have abundant nutrition, and organ growth is only limited by the intrinsic program. Worker-destined larvae have a shortage of nutrition that restricts growth and influences metabolic parameters accordingly. In contrast, the Instruction model proposes that the RJ has a compound (or compounds) that instruct the development of queens [13–15]. In support of the Instruction model, research over the past decades has attempted to identify a single compound from RJ [12, 14] that can determine queen development.

A recent study provided evidence that the protein royalactin has queen-determining activity [15]. However, follow-up experiments in another laboratory were unable to repeat these results [7], questioning the existence of a single determinant for queen development [4]. Gradually increasing the sugar levels of WJ and altering the composition of RJ-containing diets produced workers, intercastes, and eventually queens [9–11, 16], but it failed to rear only queens. The more continuous caste characteristics resulting from different feeding regimes [17] have been proposed in support of the Nutrition/Growth model. The RJ and the WJ produce different reaction norms of the general developmental program that determines the caste polyphenism. An alternative explanation is that the essential higher sugar levels for queen-destined larvae are a secondary effect and reflect the higher energy requirements for the faster and larger-growing queen organs of an otherwise instructed queen program. The rearing of larvae at day 5 in queenless colonies yielded bees with ovariole numbers that were discontinuous (either more worker or queen-like distributed), while other queen and worker traits were either absent or present in a noncorrelated fashion [18], suggesting two distinct states of the developmental program and the possible existence of regulatory switches [19].

One possible mechanism by which nutrients are sensed by bee larvae is the insulin/IGF signaling (IIS) and target of rapamycin (TOR) pathways, which link the abundance of nutrition with worker and queen differential gene expression [20–23]. Indeed, nutritional input can also influence growth and metabolic programs via the IIS and TOR pathways in mammals and other insects [24–26]. However, whether regulation of the IIS and TOR pathways drives caste differentiation or whether the regulation is a response to the activation of a queen developmental program is currently unknown. Consistent with the faster and larger growth of queens, gene expression studies have revealed the up-regulation of physiometabolic genes in queens, reflecting their higher metabolic rate [27, 28]. Chromatin modifications and DNA methylation analyses have indicated distinct epigenetic states in worker- and queen-destined larvae, suggesting another level of regulatory control associated with caste-specific gene expression [29–31].

Here, we explored whether nutrition is the only factor directing size polyphenism and whether further genetic instruction from the sex determination pathway is required. To do so, we introduced a method to screen mutations directly in worker bees using the clustered regularly interspaced short palindromic repeats/CRISPR-associated protein 9 (CRISPR/Cas9) technique.

## Results

### Worker nutrition is not a general driver for the reduced size of reproductive organs

According to the Nutrition/Growth model, nutrition is the only driver of reduced reproductive organ size, the most prominent trait in caste development. Males, like queens, receive high amounts of sugar during larval development [32] and develop large reproductive organs unlike sterile worker bees. Gradually increasing the sugar levels of WJ produces intercaste development [9, 10, 16]. Hence, if a shortage of nutrition in the worker diet (and reduced sugar levels) is the only driving component, we would expect that this diet would also mediate the size reduction of reproductive organs in males.

**Fig 1:**
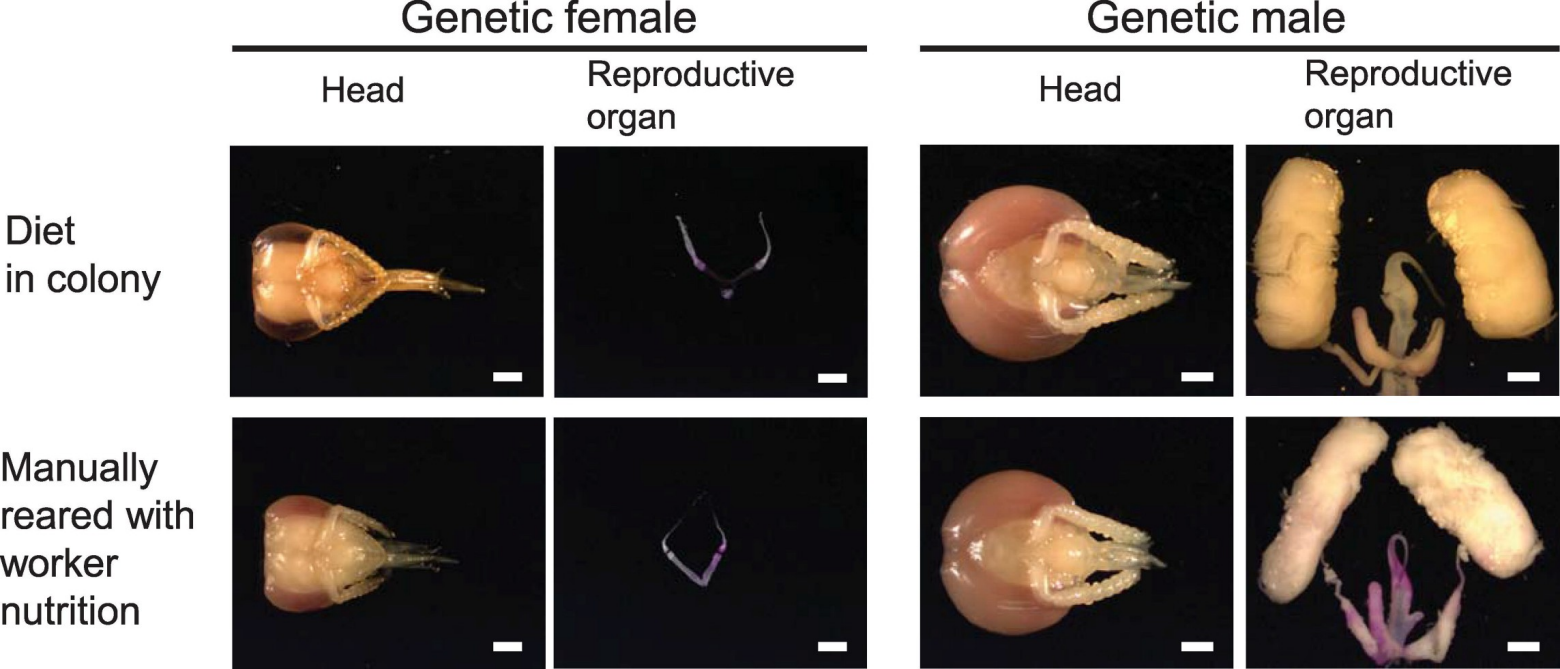
Reproductive organ and head phenotypes of females and males reared on worker nutrition in the laboratory and in the colony. Scale bar = 1 mm.

We manually reared genetic females and males on worker nutrition [16, 33] and compared their phenotypes with those of workers and genetic males reared in the colony (Fig 1 and S1 and S2 Tables). The reproductive organs of genetic female bees raised on worker nutrition either inside the colony (*n* = 14) or manually outside (*n* = 15) were equivalent in size (Fisher’s exact test, df = 1, *P* = 1). In both laboratory- and colony-reared genetic females, there were few ovarioles, and the size of each ovary was small compared with the size of the heads (Fig 1 and S1 Table). This contrasts with the large ovaries of the female larvae fed a queen diet in the hive (queens alone cannot be consistently reared under laboratory conditions [7]; see Fig 4A and 4B as an example of a queen phenotype). This result indicates that our manual feeding regime mirrors the effect of a worker diet in the hive [16, 33]. To examine whether only the balance and amount of nutrition (low amount of sugar) determine small reproductive organs, we reared genetic male larvae on worker nutrition in the laboratory and compared these with males that received high amounts of sugar in the colony [32]. Genetic males that were reared on the worker nutrition diet had large male reproductive organs (Fig 1 and S2 Table). They were equivalent in size (*n* = 20) to the males obtained from the colony (*n* = 8) that were reared on drone nutrition (Fisher’s exact test, df = 1, *P* = 1). These results indicate that worker nutrition (and a shortage of sugar) is not the only requirement for the size polyphenism, suggesting input from the sex determination pathway.

### Somatic mutational screening in reared bees

We next established a method that enables the mutational screening of sex-determining genes directly in worker bees using the CRISPR/Cas9 method [34–36]. Following traditional mutant approaches, we would need to produce mutant queens and drones that need to be crossed to generate double-mutant worker bees. If we could mutate all nuclei in the embryo, we would be able to directly rear mutated worker bees without maintaining colonies and performing crossings. To examine whether we could mutate worker bees entirely using the CRISPR/Cas9 method, we tested different embryonic injection conditions. To determine the robustness of this approach, we studied at least two sites for three genes, the *doublesex* (*dsx*), *fruitless* (*fru*), and *loc552773* genes (S1 Fig). Only the *dsx* gene was used later on for phenotyping. We injected into the anterior embryos of very young female embryos (0 to 1.5 hours after egg deposition) [37]. We tested a set of single guide RNAs (sgRNAs; S3 Table) at different concentrations and observed that we repeatedly mutated each injected embryo.

**Fig 2:**
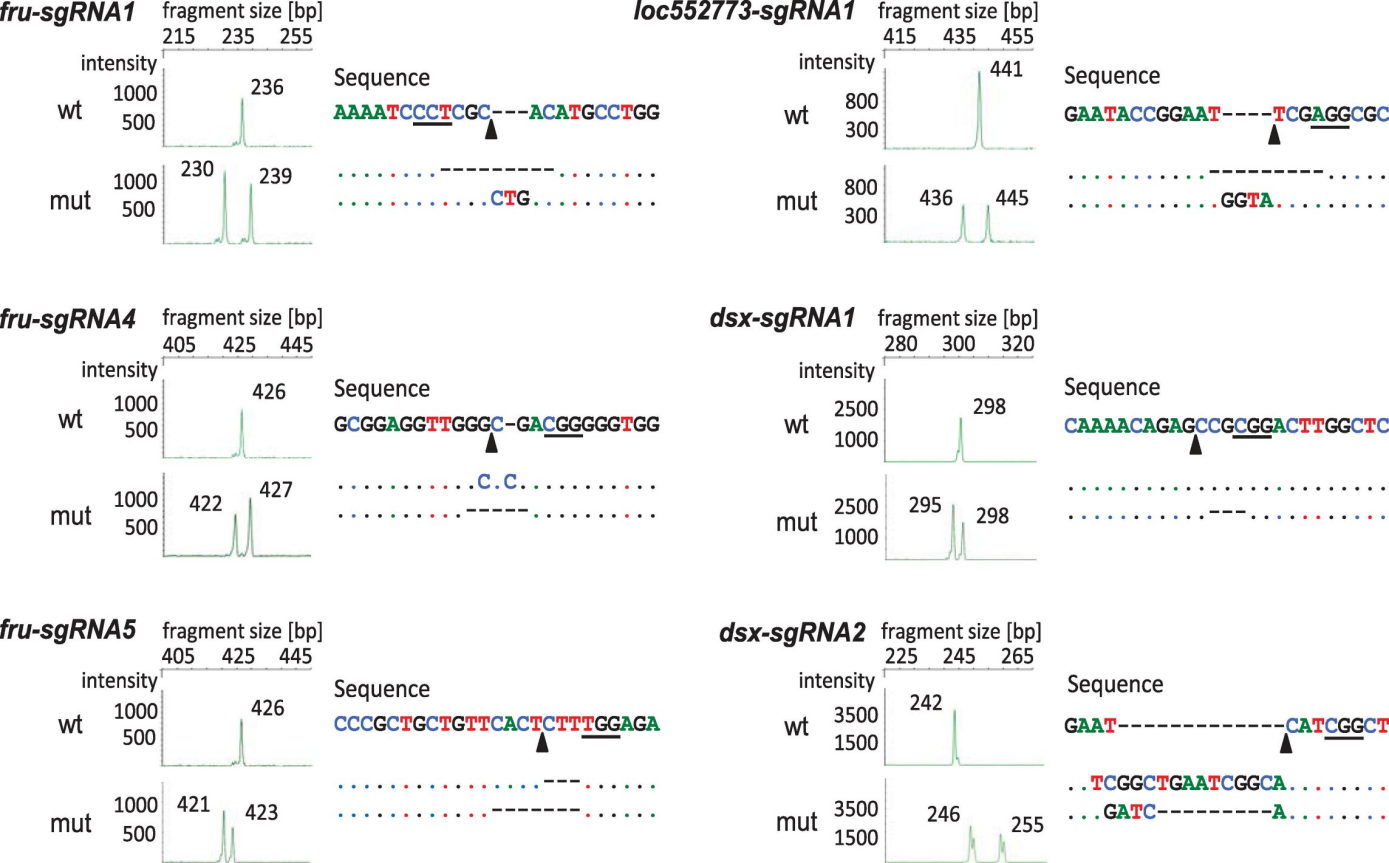
Examples of FL and nucleotide sequence analyses of the targeted genomic sites of single bees using the efficient CRISPR/Cas9 method. FL analysis is presented on the left, and the nucleotide sequences are presented on the right for single bees. Examples of WT alleles and mutated sequences are shown. The cleavage site of the Cas9 protein is indicated with arrows. The PAM site (the essential targeting component for CRISPR/Cas9) is underlined in the nucleotide sequence. Dashes indicate deletions. CRISPR/Cas9, clustered regularly interspaced short palindromic repeats/CRISPR-associated protein 9; FL, fragment length; mut, mutated sequences; PAM, Protospacer adjacent motif; WT, wild type.

The fragment length (FL) and sequence analyses of the amplicons in larval stage 1 larvae revealed that up to 100% of the *fru* and *dsx* and 60% of the *loc552773* target embryos were mutated (Tables 1 and S4 and S5 and Fig 2). The wild-type (WT) allele was consistently not detected in 30 of the 39 mutated larvae (77%), suggesting that all nuclei (to the level of detection) and both alleles in the larvae were mutated (generating double mutants). More than two mutated sequence variants were detected in a single larva (3%), while singly mutated sequences together with the WT allele were detected in 8 larvae (20%) (S4 and S5 Tables). Indels occurred most frequently between the 5 bp to 1 bp range, with 44% of mutations being deletions and 20% resulting in insertions (S5 and S6 Tables). All mutations occurred at the designated target site. Therefore, our results on the adjustments demonstrate that nearly 80% of the injected embryos had mutations on both alleles (double mutants) affecting the bee entirely (absence of mosaicism). This high proportion enabled us to screen for mutant effects of the sex-determining genes directly in the injected bees.

**Table 1:** Frequency of the mutated honeybee larvae based on FL analyses at single base-pair resolution of the amplicons.

**Treatment**	**pg of Cas9 mRNA per embryo**	**pg** **of sgRNA** **per embryo**	**No. of surviving embryos 24 h after injection**	**No. (%) of hatched L1 larvae**	**No. of genotyped larvae**	**No. of larvae with length variant**^1^	**Efficiency of mutagenesis**^2^
**fru-sgRNA1**	800	29.2	105	10 (10%)	8	2	20%
**fru-sgRNA2**	400	14.6	467	72 (15%)	7	6	86%
**fru-sgRNA1**	240	8.8	78	2 (3%)	2	2	100%
**fru-sgRNA4**	400	14.6	125	3 (2%)	3	3	100%
**fru-sgRNA5**	400	14.6	98	10 (10%)	10	10	100%
**loc-sgRNA1^3^**	400	14.6	93	7 (8%)	5	3	60%
**loc-sgRNA2**	400	14.6	102	32 (31%)	28	1	4%
**dsx-sgRNA1**	400	5.5	52	1 (2%)	1	1	100%
**dsx-sgRNA1**	400	3.7	93	5 (5%)	4	1	25%
**dsx-sgRNA2**	400	5.5	178	2 (1%)	2	2	100%
**dsx-sgRNA2**	400	3.7	89	5 (6%)	5	5	100%
**dsx-sgRNA2**	400	0.7	82	21 (26%)	19	3	16%
**H**_**2**_**O**	-	-	48	27 (56%)	11	0	0%
**Uninjected**	-	-	65	55 (85%)	19	0	0%

^1^Fragments differed in length compared with fragments isolated from 7 nontreated (WT) larvae.

^2^Relative ratio of the number of mutant larvae to the number of all larvae.

^3^Targeted the gene *loc552773*.

**Abbreviations:** Cas9, CRISPR-associated protein 9; FL, fragment length; pg, picogram; sgRNA, single guide RNA; WT, wild type.

### The *feminizer* gene is required for small size polyphenism

**Fig 3:**
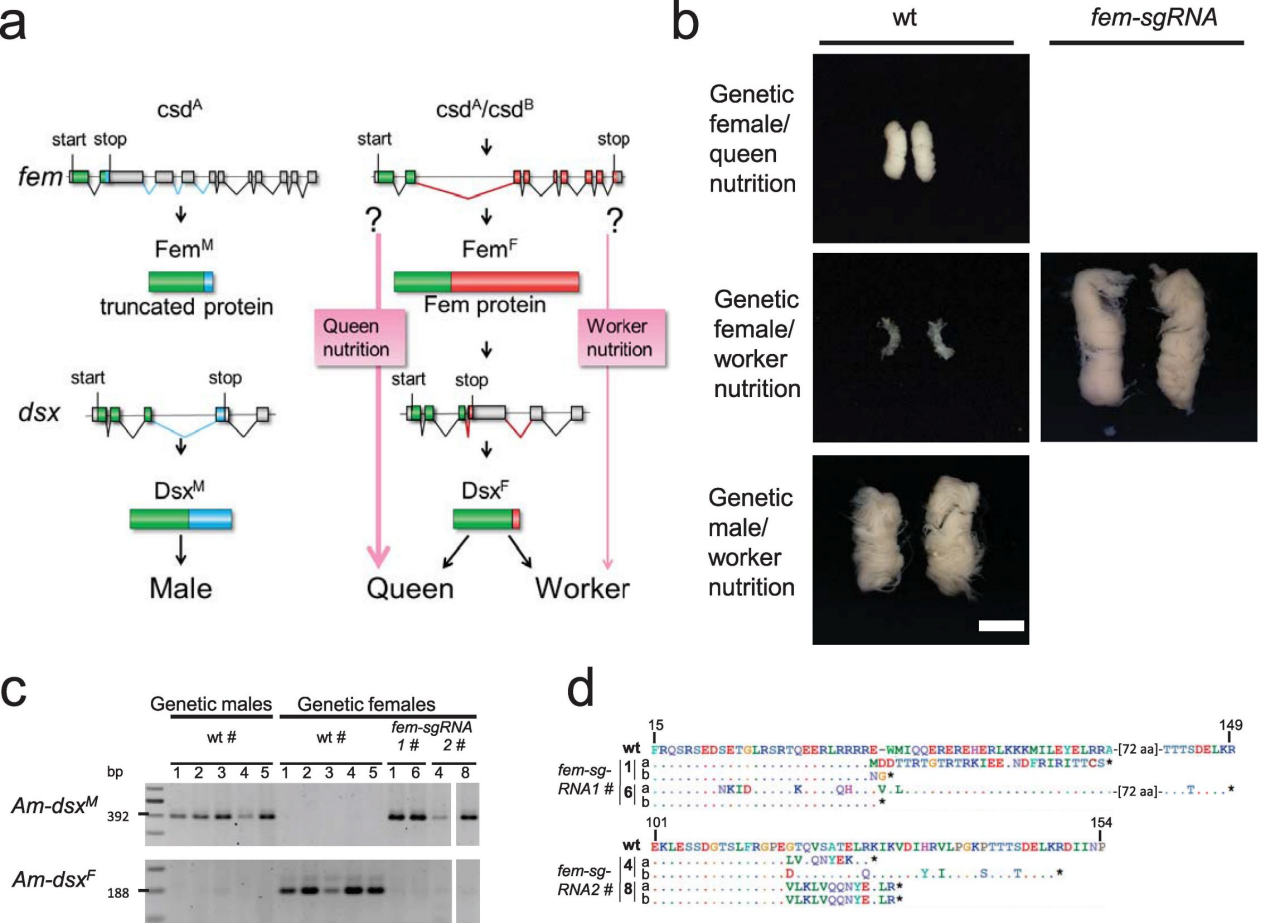
Size polyphenism of gonads in genetic females at larval stage 5 that were double mutants for the *fem* gene. (a) Model of the known components of the sex-determining pathway in honeybees with nutritional differences in females. (b) Gonad development at larval stage 5. (Right) A pair of large gonads (male type) from *fem sgRNA2*-treated genetic females reared on worker nutrition. The gonads display densely packed layers of folded testioles, similar to those observed in haploid males (WT males). (Left) Pairs of small gonads (female type) from WT workers and genetic female bees reared on worker nutrition. A WT large queen ovary from a queen reared in a colony on queen nutrition. A large WT testis of a haploid male manually reared on worker nutrition. (c) Male *dsx* (*dsx*^*M*^) and female *dsx* (*dsx*^*F*^) transcripts in mutated genetic females with male phenotypes (*fem-sgRNA1* or *fem-sgRNA2*). Male and female transcripts were separately amplified by RT-PCR [64], and the male and female fragments of each single bee were resolved via agarose gel electrophoresis. Numbers indicate different control and mutated bees. (d) Deduced amino acid sequences from sequenced amplicons of the *fem* gene at the designated CRISPR/Cas9 cleavage sites for the four worker nutrition-reared genetic female larvae with large gonads of the male type. Stars indicate premature translation stop codons. Numbers indicate different mutated bees. Scale bars, 1 mm. CRISPR/Cas9, clustered regularly interspaced short palindromic repeats/CRISPR-associated protein 9; *dsx*^*F*^, female *dsx*; *dsx*^*M*^, male *dsx*; RT-PCR, reverse transcription PCR; sgRNA, single guide RNA; WT, wild type.

To examine whether the *feminizer* (*fem*) gene is required for small size polyphenism, we mutated the gene in genetic females and reared them with worker nutrition. The *fem* gene instructs female development and maintains the female signal during development, as revealed from *fem* interference RNA (RNAi) knockdown and mosaic studies using a non–worker-specific diet for bee rearing [19, 38]. The Fem protein is encoded by female-specific spliced *fem* transcripts but not the male spliced variant, which harbors an early stop codon [19] (Fig 3A). The female splicing of *fem* is directed by the c*omplementary sex determiner* (*csd*) gene when the genotype is heterozygous (Fig 3A) [39]. If the *fem* gene is required for small size polyphenism, we would expect that worker nutrition cannot drive size reduction when *fem* is inactive. If the *fem* gene is dispensable, worker nutrition would drive size reduction even when the *fem* gene is inactive. We induced mutations at two target sites in the first half of the female open reading frame (ORF) of the *fem* gene with *fem-sgRNA1* and *fem-sgRNA2* (S1 and S2 Figs) and reared genetic females with worker nutrition to larval stage 5. Fifteen percent of the reared and injected genetic females (heterozygous for the *csd* gene; S7 Table) were double mutants for nonsense mutations as revealed from the sequenced amplicons (S8 Table and S2 Fig). These double mutants (*n =* 4) had large gonads (Fig 3B and 3D) compared with the small gonads of WT genetic females reared on worker nutrition (*n* = 38, Fisher’s exact test, df = 1, *P* < 0.001, S9 Table). The large gonads in the mutants were of the male type. They consisted of packed layers of multiple testioles of the same size as those of the males reared on worker nutrition (Fig 3B) and those of the males in the colony (Fig 1). The female *fem* mutants lost the female *dsx* transcript and only displayed the male *dsx* transcript (Fig 3C), demonstrating that the mutant bees entirely switched in their development from female to male identity. These results indicate that *fem* is required for size polyphenism or that size polyphenism relies on the intrinsic program of the female differentiating tissue induced by *fem*.

### *dsx* is dispensable for small size polyphenism

**Fig 4:**
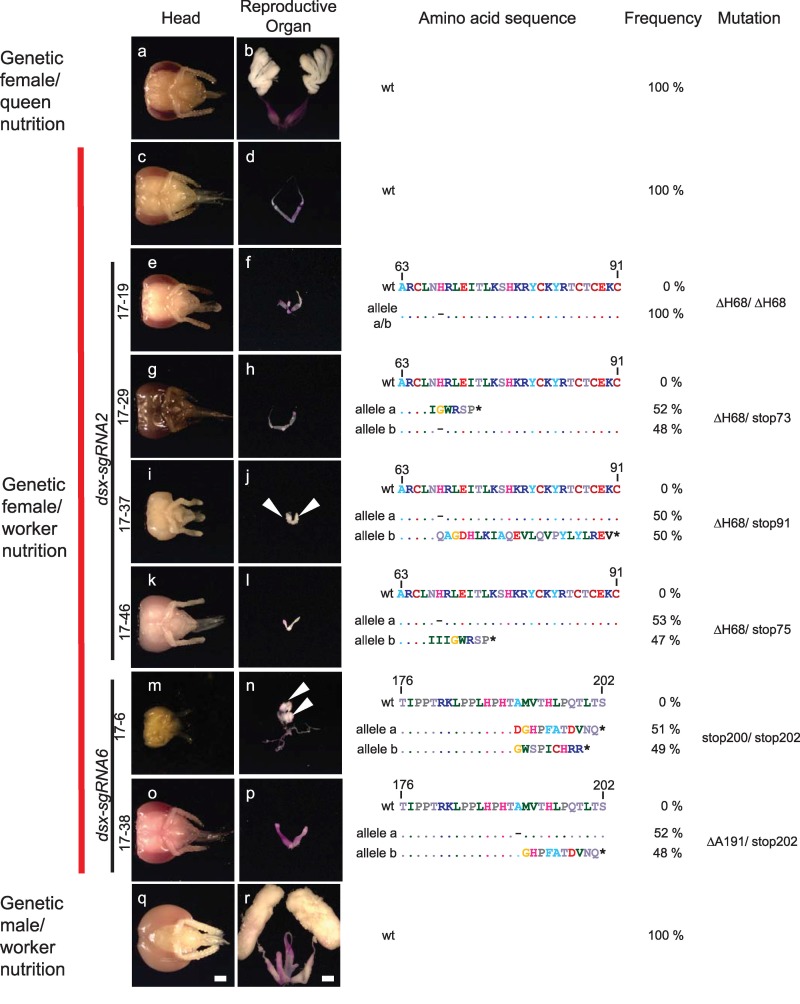
Size polyphenism of the reproductive organs in genetic female double mutants for the *dsx* gene. Pictures of the head and internal reproductive organs of mutated and WT control bees are shown on the left, while the genotypes at the *dsx* locus with the deduced amino acid sequences are displayed on the right. Mutated and control genetic females and males were reared on worker nutrition. Queens were reared on the queen diet in a colony (we cannot mimic queen rearing in the laboratory). The WT amino acid sequence is shown above the detected alleles for comparison. (a, b) WT genetic female reared on queen nutrition (RJ) in the colony. (c, d) WT genetic females manually reared on worker nutrition. (e–l) Genetic females reared on worker nutrition that were double mutants for *dsx* via the *dsx-sgRNA6* (note that a small part of the worker bee head 17–39 [picture i] is missing due to the dissection process). (m–p) Genetic females reared on worker nutrition that were double mutants for *dsx* via the *dsx-sgRNA2*. (q, r) Genetic males manually reared on worker nutrition. Organs were stained with aceto-orcein (reddish coloring) to facilitate the dissection process. Testis tissues are marked with arrows. Scale bar, 1 mm. Dashes in the sequence indicate deletions, and stars illustrate early translational stop codons. RJ, royal jelly; WT, wild type.

To examine the role of female *dsx* on size polyphenism of the reproductive organ, we mutated the *dsx* gene in genetic females and reared them on worker nutrition. If *dsx* is dispensable, we would expect small size polyphenism even when *dsx* activity is compromised. In *Drosophila melanogaster*, the *dsx* gene essentially controls, beside the reproductive organs, all aspects of somatic sexual differentiation [40, 41], and it controls at least reproductive organ development in other insects that belong to different insect orders, including hymenopteran insects [42–45]. The *dsx* transcripts in honeybees are sex-specifically spliced by the presence of the Fem protein in females and the absence of the Fem protein in males [19] (Fig 3A). The sexual splice variants encode a transcription factor with an intertwined zinc-containing DNA binding (DM) domain and male- and female-specific termini at the carboxyl end [46–50]. We mutated the *dsx* gene at two target sites in the non–sex-specific expressed N-terminal portion. *dsx-sgRNA2* targeted the DM domain, whereas *dsx-sgRNA6* targeted a downstream region in exon 3 (S1 Fig). The treated genetic females were reared on worker nutrition and were examined for morphological changes of the reproductive organ and head. Genotyping of the mutated bees with morphological changes via next-generation sequencing (NGS) of the amplicons revealed that they were regularly double mutants with an approximate ratio of 1:1, suggesting that the mutations belong to the two chromosomes of the diploid set. If we detected more than two sequence variants per bee, we excluded these bees from further phenotype analysis as they were genetic mosaics (e.g., a mosaic of differently mutated cells). Eleven (17%) of the adult or pupal bees had intersex morphology in the reproductive organs compared with the WT genetic females (S10 Table). No effect was observed for the heads. The following mutations were the most common ones in the genetic females: (i) different nonsense mutations that introduced new stop codons at various positions in exons 2 and 3, (ii) deletions of amino acids in the DM domain mainly the histidine codon at amino acid position 68 (ΔH68), and (iii) deletion of the alanine codon (ΔA191) at amino acid position 191 (Fig 4 with the deduced amino acid sequences and S3 Fig with the detected nucleotide sequences). The ΔH68 mutation removes a histidine of the DM domain that is essential for the zinc binding and DM domain functions [47, 51] and that is conserved between vertebrates and invertebrates (S4 Fig). The intersex reproductive organs were all of the same small size (*n =* 11) as the worker reproductive organs in WT genetic females that were manually reared on worker nutrition (*n =* 17, Table 2, Fisher’s exact test, df = 1, *P* = 1). The small intersex reproductive organs displayed either male gonads with poorly or non–sex-specifically differentiated duct systems (*n =* 4), as observed in stop200/stop202 and ΔH68/stop91 genetic females (arrows in Figs 4 and S5). The potentially earlier developmental stage of some of these mutant bees cannot explain why these male-like gonads are so small because the distinct size differences of male and worker gonads are also present at earlier pupal stages (S6 Fig). In other cases, the reproductive organs were underdeveloped (*n =* 7), and the oviducts were consistently misshaped while the ovarioles were repeatedly missing, as identified in ΔH68/ΔH68, ΔH68/stop73, ΔH68/stop75, and ΔA191/stop202 genetic females (Figs 4 and S5). The heads of the mutant genetic females with intersex reproductive organs were all of worker type (*n =* 11, Fig 4 and S10 Table), suggesting that *dsx* is not required for sexual development of the head. The results of the consistently small, intersex reproductive organs with varying degrees of masculinization suggest that *dsx* is not required for size polyphenism.

**Table 2:** The size of the intersex reproductive organs in genetic females double mutant for *dsx* and reared on worker nutrition.

Sex	Nutrition	Genotype	Reproductive organ	Numbers	Size of reproductive organ^a^	
					<2.5 mm; <0.7 times the size of the head width	>6 mm; >1.2 times the size of the head width
**Genetic female**	Manually reared on worker nutrition	*dsx* double mutants	Intersex	11	11 (100%)	0 (0%)
		WT	Worker	17	17 (100%)	0 (0%)
	Queen diet in colony	WT	Queen	3	0 (0%)	3 (100%)
**Genetic male**	Manually reared on worker nutrition	WT	Male	16	0 (0%)	16 (100%)

^a^Length between the fused left and right part of the reproductive organ to its end in the sagittal plane.

**Abbreviation:** WT, wild type.

## Discussion

**Fig 5:**
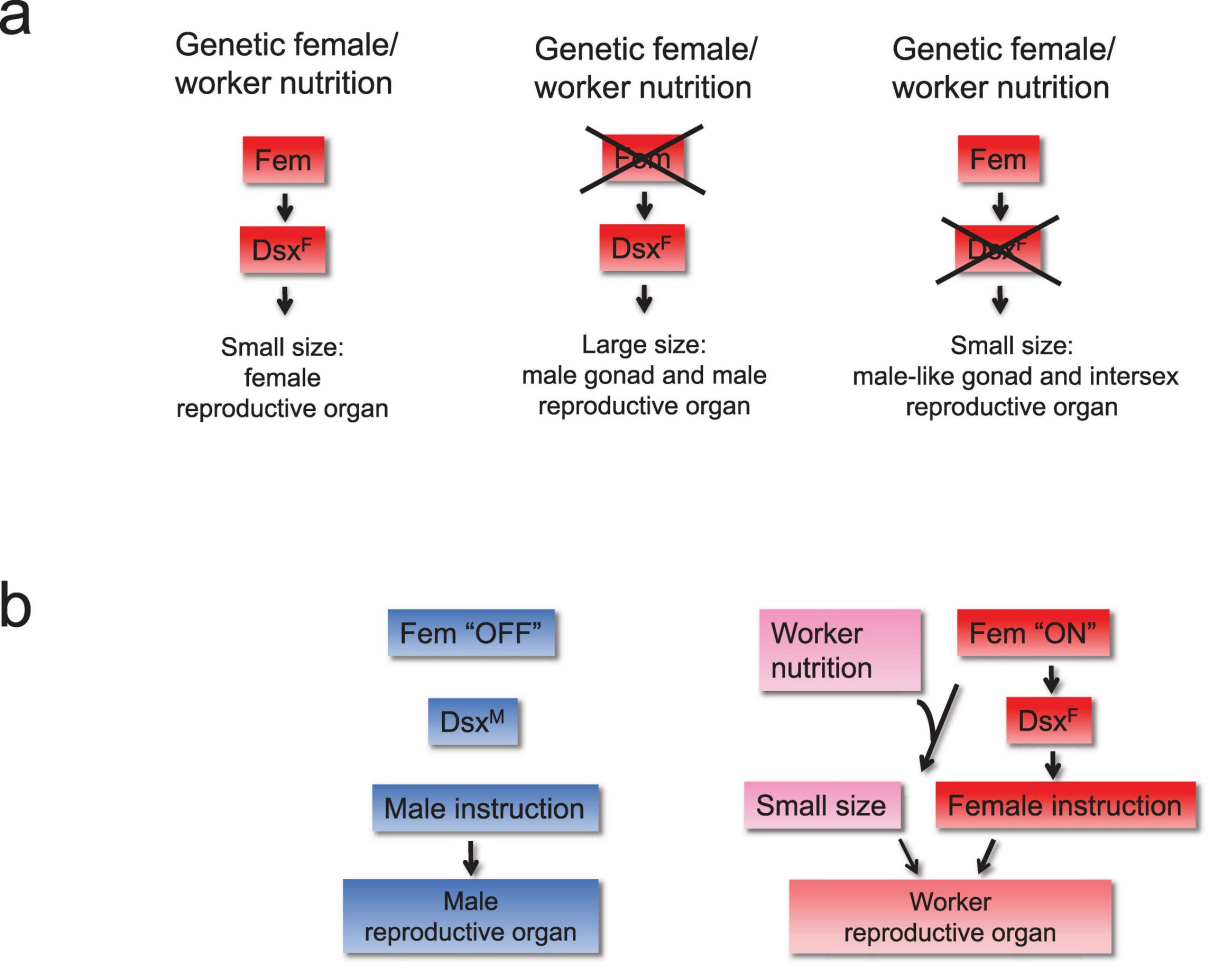
The role of the sex-determining genes *fem* and *dsx* in size polyphenism. (a) Schematic presentation of the mutant effects of *fem* and *dsx* gene on size polyphenism. Genetic female bees reared on worker nutrition produce only small reproductive organs. Genetic females with a mutant *fem* gene show no small size polyphenism of reproductive organs. Genetic females that have a mutated *dsx* (operating downstream of *fem*) do show size polyphenism of the intersex reproductive organ and male-like gonads. Thus, we conclude that the *fem* gene is required for the small size polyphenism. Crosses mark the genes that we compromised using CRISPR/Cas9-induced mutations. (b) The role of the *fem* gene for caste development. The gene products of the sex determination pathway (Fem, Dsx^F^, Dsx^M^) are shown in red (female) and blue (male) boxes. The nutrition-mediated process is shown in pink. Arrows indicate regulatory relationships. CRISPR/Cas9, clustered regularly interspaced short palindromic repeats/CRISPR-associated protein 9.

Caste polyphenism in honeybees is determined by different nutrition with the size of the reproductive organ as an important trait. Most studies suggest that the balance and amount of nutrition (Nutrition/Growth model) drive the size polyphenism between queens and workers. Our genetic and rearing results now suggest that the response to nutrition relies on a genetic program that is switched on by the *fem* gene. The genetic females with a mutant *fem* gene show large size reproductive organ (large polyphenism), while WT genetic females (Fig 5A) reared on the same worker nutrition have only small reproductive organs (small polyphenism). Genetic females that have a mutated *dsx* gene (operating downstream of *fem*) do show small reproductive organs (small size polyphenism; Fig 5A). *dsx* mutants produce intersex reproductive organs and male-like gonads that are all of small size, demonstrating that small size does not rely on female development of the tissue. The small size polyphenism also did not result from *dsx* malfunction because (i) small phenotypes were consistently observed irrespective of the different degrees of *dsx* malfunctions we introduced by missense and nonsense mutations (Fig 4) and (ii) *dsx* mutations in other insects did not influence the size of the reproductive organs [42, 52, 53]. Thus, the results together suggest that the *fem* gene is required for the small size polyphenism. We conclude that the *fem* gene must be switched “ON” so that size polyphenism can be executed (Fig 5B). The essential role of the *fem* gene in small size polyphenism assigns a further key function to the *fem* gene. Previous studies demonstrated that the *fem* gene is also required to (i) induce entire female development in response to the primary signal *csd* [19, 38] and to (ii) maintain the female signal during development via a positive regulatory feedback loop [19]. Whether *fem* also instructs the large size polyphenism of queens needs further functional testing once a queen-only rearing protocol has been developed for the laboratory [7].

The genetic instruction via the *fem* gene provides an entry point to dissect nutrition-mediated control. Our results suggest that the *fem* gene switches “ON” the machinery that is required for sensing the worker nutrition and for implementing the size polyphenism. Because the *fem* gene encodes a serine arginine rich (SR)-type protein, the direct targets of the *fem* gene involved in size polyphenism may also be activated by sexual splicing. The *fem*-controlled candidate genes can be functionally tested by determining whether they affect the size polyphenism. The function will be directly tested in mutated genetic females as demonstrated in this study.

Our mutant analysis further demonstrate that *dsx* controls female differentiation of the reproductive organs. The mutant honeybee phenotypes of the reproductive organs in honeybees yielded similar phenotypes as in female *D*. *melanogaster*. Female *dsx*-mutant fruit flies have reproductive organs of varying intersex phenotypes. The organs are often underdeveloped with occasionally developed ovaries, but are frequently of the “male type” [52, 54, 55]. The internal duct system can develop into a mixture of female/male or single poorly differentiated ducts [52]. RNAi-mediated knockdown studies on the beetle *Tribolium molitor*, housefly *Musca domestica*, and sawfly *Athalia rosae*, as well as conditional expression and CRISPR/Cas9 experiments on the silkworm *Bombyx mori*, have revealed sex-related effects on internal reproductive organ development [42–46, 53]. Our results support a conserved role for *dsx* in the sexual development of the reproductive organ. However, in honeybees there is a nutrition-driven size control of reproductive organ development that operates upstream of or in parallel with *dsx-*regulated sexual development.

The first CRISPR/Cas9-induced morphological mutants in honeybees introduced a new genetic screening method for worker bees. We efficiently induced mutations in injected embryos using the CRISPR/Cas9 method [34, 35] and directly screened for somatic mutations in the reared honeybees (somatic mutation approach). Up to 100% of the embryos were mutated, and mosaicism among the mutated embryos was rare (<10%). The previous studies in honeybees using CRISPR/Cas9-induced mutations report on 1 out of 2 queens with only 12% and 2 out of 4 queens with only 5% and 10% mutant drone offspring, suggesting that the previously published method has a substantial lower rate and produced strong mosaicism in the queens [36, 56]. These previous studies generated no worker bees that would require further crossing experiments. With very early embryonic injections [37] and a selection step to identify the most efficient sgRNAs and Cas9 concentrations, we generated mutation rates of up to 100% and no mosaicism in worker bees directly. The rearing of the mutated embryos to worker bees was performed under controlled conditions in the laboratory [16, 33]. This required no rearing of queens and drones and crossing experiments. The procedure was demonstrated for mutations at two target sites for two genes and their morphological changes (Figs 3 and 4). The absence of mosaicism and completeness of mutagenesis of this procedure were shown by the results that most mutated bees lost the WT allele (they were double mutants; Figs 2, 3D and 4) and that double *fem* nonsense mutations produced an entire female to male switch, including *dsx* splice products (Fig 3C). This somatic mutation approach does not require further crossing experiments and laborious maintenance of hundreds of colonies and therefore offers the prospect of larger genetic screens in honeybees. In other insects in which somatic mutation approaches have been applied [57, 58], the adults were genetic mosaics in which parts of the butterfly wing were WT while other parts were mutated. Enhancing the efficiency of mutagenesis can thus provide an opportunity for somatically testing gene functions in insects that are not yet genetically trackable.

## Methods

### sgRNA and mRNA syntheses

*Cas9* mRNA was synthesized from the *Cas9* gene [59] (Vector MLM3613, ID #42251, Addgene, Cambridge, MA) using a linearized plasmid via the T7 promoter and the mMESSAGE mMACHINE Kit (Ambion, Darmstadt, Germany). mRNAs were polyadenylated using the Poly(A) Tailing Kit (Ambion). Target sites for the sgRNAs were identified via Optimal Target Finder software (http://tools.flycrispr.molbio.wisc.edu/targetFinder/). sgRNAs were 20 nt long with a G nucleotide at the 5´ end. sgRNAs with no off-target effects or with at least three nucleotide mismatches to alternative target sites were selected. sgRNAs were generated via PCR without a template using two overlapping oligonucleotide sequences containing the sequence of the T7 RNA polymerase transcription start site, the gene-specific target site and the Cas9 protein-binding site. sgRNAs were synthesized using a RiboMax Kit (Promega, Madison, WI) according to the manufacturer’s instructions. RNAs were purified using the MEGAclear Kit (Ambion).

### Microinjection and rearing

Embryos were microinjected 0 to 1.5 hours after egg deposition [19, 37, 60] using 53-mm injection pipettes (Hilgenberg, Malsfeld, Germany). Cas9 mRNA or protein (New England Biolabs, Ipswich, MA) was applied at 400 to 2,000 ng/μl and mixed with sgRNAs using a molar ratio of 1:2 to 1:0.75. The number of injected embryos that hatch can vary greatly between experiments and sgRNAs (5% to 40%). Rearing was performed using a mass rearing technique for the worker bees [16, 33]. Freshly hatched larvae were provisioned only once with the worker larval diet (50%–53% RJ, 4% glucose, 8% fructose, 1% yeast extract, and 30%–34% water), approximately 120 to 170 mg of which was consumed [16, 33]. The larvae were incubated at 34°C and 90% humidity until the larval stage 5 or to adults. For pupal rearing we also used a slightly different diet for larvae at stage 5 (50 mg diet 2 [50% RJ, 12% fructose, 6% glucose, 2% yeast extract, and 30% water]).

### DNA preparation, RNA isolation, and cDNA synthesis

For genotyping, genomic DNA was isolated from freshly hatched L1 or L5 larvae [61] using the peqGOLD Tissue DNA Mini Kit (VWR, Darmstadt, Germany). RNA was isolated using the TRIZOL method (Thermo Scientific, Braunschweig, Germany), and cDNA was synthesized using the RevertAid First Strand cDNA Synthesis Kit (Thermo Scientific). Second-strand cDNA synthesis was performed by adding 10 μl of 10× DNA Polymerase Buffer, 40 U DNA Polymerase I, 0.8 U Ribonuclease H, and 65.68 μl of dH_2_O to 20 μl of the cDNA first-strand synthesis product. Double-stranded cDNA was purified using the EZNA Cycle Pure kit (Omega Bio-Tek Inc., Norcross, GA).

### PCR, sequencing, and FL analysis

All mutant bees were genotyped by sequencing the amplicons of the targeted site. PCR amplifications were performed using standard conditions [62] and GoTaq polymerase (Promega). Oligonucleotide sequences were synthesized at Eurofins (Ebersberg, Germany). Amplicons were either cloned and sequenced (Sanger sequencing [Eurofins]) or sequenced via NGS. NGS index PCR was performed using the Nextera XT Index Kit (Illumina, San Diego, CA), and purification of the Index PCR products was performed using Agencourt AMPure XP beads (Beckman Coulter, Brea, CA). NGS was performed on an Illumina MiSeq system using the MiSeq Reagent Kit version 2 (500 cycles; Illumina), generating 800,000 paired-end reads with a read length of 2 × 250 bp, resulting in approximately 15,000 paired-end reads per sample. We removed contamination by removing sequences that were less frequent than 5%. The FLs of hexachlorofluorescein (HEX)-labeled amplicons were determined using an ABI 3130XL Genetic Analyzer (Applied Biosystems, Darmstadt, Germany) and Peak Scanner software (Thermo Scientific). For the *fem* mutants, we conducted fragment and sequence analysis on the amplicons of the cDNAs to ensure that the many *fem*-related sequences observed at the genomic *fem* locus (derived from duplication events) [63] were not amplified.

## Supporting information

S1 Fig Genes and targeted genomic sites. Genomic organization of the genes *fru* (a), *loc55277*3 (b), *dsx* (c), and *fem* (d) with the designated sgRNA target sites (black arrows). Boxes indicate exons. If genes transcribe sexual splice variants, they are presented. Green boxes indicate common, red the female-specific, and blue the male specific ORF of the sexual transcripts. *dsx*, *doublesex*; *fem*, *feminizer*; *fru*, *fruitless*; ORF, open reading frame; sgRNA, single guide RNA.
(PDF)

S2 Fig The nucleotide sequences of the *fem*-mutated genetic females that were reared on worker nutrition and that have large-sized gonads of the male type. (a) Diagrams of the FL analysis for each of the 4 individuals and WT worker bee examples. (b) The nucleotide sequences. We conducted fragment and sequence analysis on amplicons of cDNA to ensure that the many *fem*-related sequences observed at the *fem* locus (derived from duplication events) [63] were not amplified. The designated binding sites of the sgRNAs are underlined. Sequence b in larvae #4 resulted from fusion of exon 3 with exon 5. The sequences in larvae #4 resulted from fusion between exon 3 and other *fem*-related sequences [63]. The WT sequences were obtained from a sample of 5 WT worker larvae (5 clones each). cDNA, complementary DNA; FL, fragment length; WT, wild type.
(PDF)

S3 Fig Genotypes of *dsx*-mutated females of Fig 4 as obtained from NGS analyses. The *dsx* WT nucleotide sequences are represented as a reference sequence. NGS, next-generation sequencing; WT, wild type.
(PDF)

S4 Fig Alignment of the amino acid sequence harboring the zink finger motifs (ZF I and ZF II) of the DM domain. The deleted conserved histidine at position 68 of the honeybee sequence (*Am*) is highlighted with an arrow.
(PDF)

S5 Fig The intersex reproductive organs of Fig 4 at higher magnification. Scale bar, 1 mm. The genetic females were double mutant for *dsx* and reared on worker nutrition. For further details, see legend of Fig 4 in the main text.
(PDF)

S6 Fig The phenotypes of worker nutrition-reared genetic females and genetic males at an early pupal stage. These females have the typical reduced reproductive organ of workers and the fully developed reproductive organs of males. Head and (a) and (c) and reproductive organ (b) and (d). Gonads were stained with aceto-orcein (reddish coloring) to facilitate the dissection process. Scale bar = 1 mm.
(PDF)

S1 Table The worker bees reared in the colony and the genetic female bees reared manually on worker nutrition. (PDF)

S2 Table The genetic male bees reared in colony and manually on worker nutrition. (PDF)

S3 Table Nucleotide sequences of the sgRNAs. Sequences complementary to the designated genomic target site are shown in bold letters. sgRNA, single guide RNA.
(PDF)

S4 Table The numbers of mutated larvae and the numbers of length-modified (different to the WT) sequences. WT, wild type.
(PDF)

S5 Table Nucleotide sequence changes detected in the mutated larvae at the designated target site. At least 10 clones for each larvae were sequenced. These nucleotide changes were consistently not observed in 7 nontreated (WT) larvae. The sequence complementary to the *sgRNAs* are underlined. sgRNA, single guide RNA; WT, wild type.
(PDF)

S6 Table The detected deletions and insertions mediated by CRISPR/Cas9 method in a sample (*n =* 25) of mutated nucleotide sequences. CRISPR/Cas9, clustered regularly interspaced short palindromic repeats/CRISPR-associated protein 9.
(PDF)

S7 Table The heterozygous, female genotype of the *csd* gene in the *fem* double nonsense mutants. (PDF)

S8 Table Large gonads of the male type in genetic females double mutant for *fem*. (PDF)

S9 Table Reproductive organ size of genetic females at larval stage 5 that were double mutant for *fem* and that were reared on worker nutrition. (PDF)

S10 Table The reared genetic females with intersex reproductive organ that were double mutant for *dsx*. (PDF)

## Abbreviations

CRISPR/Cas9: clustered regularly interspaced short palindromic repeats/CRISPR-associated protein 9
csd: complementary sex determiner
dsx: doublesex
fem: feminizer
FL: fragment length
fru: fruitless
HEX: hexachlorofluorescein
IIS: insulin/IGF signaling
NGS: next-generation sequencing
ORF: open reading frame
PAM: Protospacer adjacent motif
RJ: royal jelly
RNAi: interference RNA
RT-PCR: reverse transcription PCR
sgRNA: single guide RNA
SR: serine arginine rich
TOR: target of rapamycin
WT: wild type
WJ: worker jelly
