# Supplementary figures and images for: A genetic switch for worker nutrition-mediated traits in honeybees
Source: PLoS Biol. 2019 Mar 21;17(3):e3000171. doi: 10.1371/journal.pbio.3000171 (PMC6428258; doi:10.1371/journal.pbio.3000171)

**a** *fruitless*

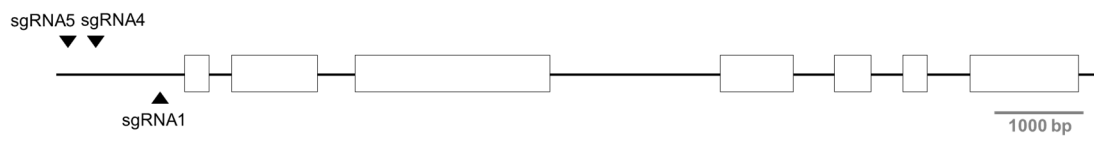

**b** *loc552773*

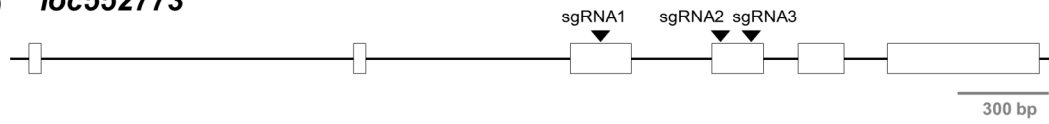

**c** *doublesex*

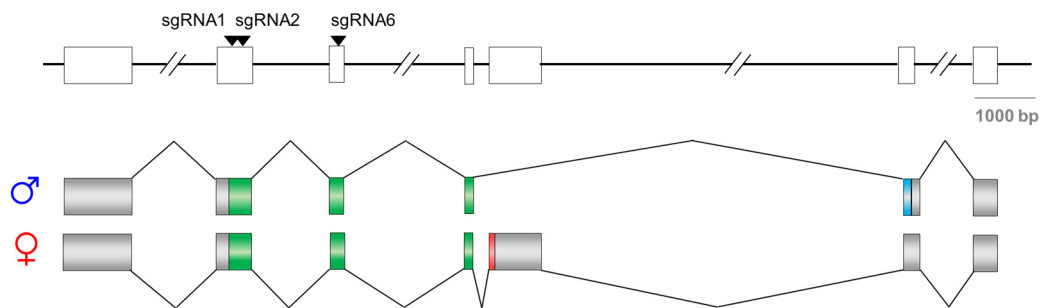

**d** *feminizer*

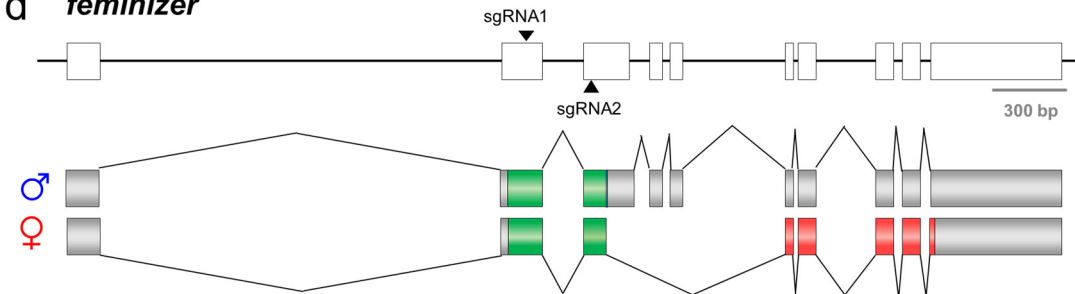

Supplement: S1 Fig — Genomic organization of the genes fru (a), loc552773 (b), dsx (c), and fem (d) with the designated sgRNA target sites (black arrows). Boxes indicate exons. If genes transcribe sexual splice variants, they are presented. Green boxes indicate common, red the female-specific, and blue the male specific ORF of the sexual transcripts. dsx, doublesex; fem, feminizer; fru, fruitless; ORF, open reading frame; sgRNA, single guide RNA. (PDF) [file pbio.3000171.s001.pdf]

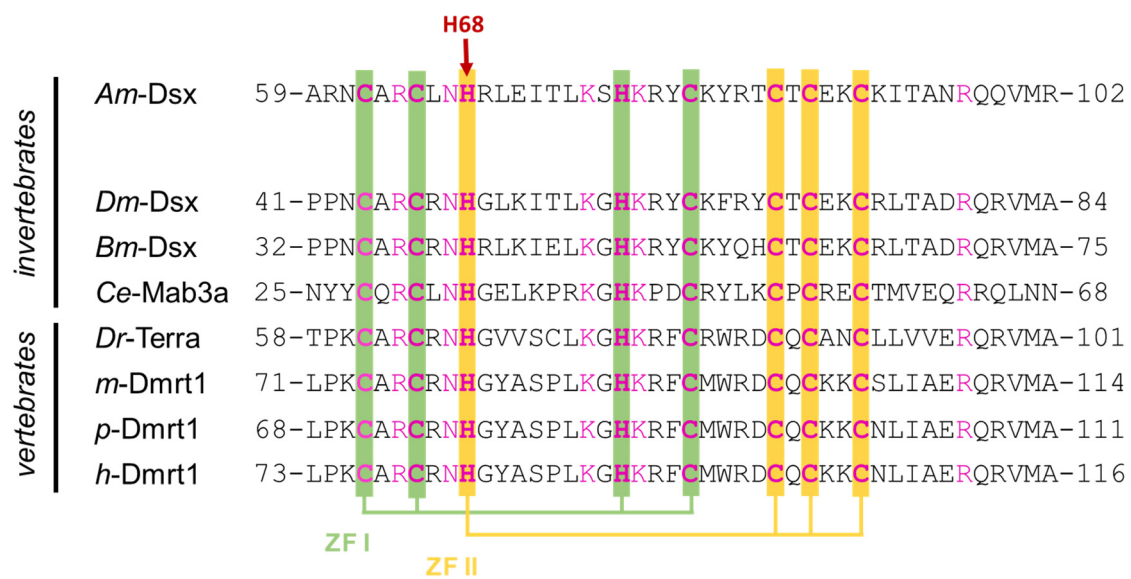

Supplement: S4 Fig — The deleted conserved histidine at position 68 of the honeybee sequence (Am) is highlighted with an arrow. (PDF) [file pbio.3000171.s004.pdf]

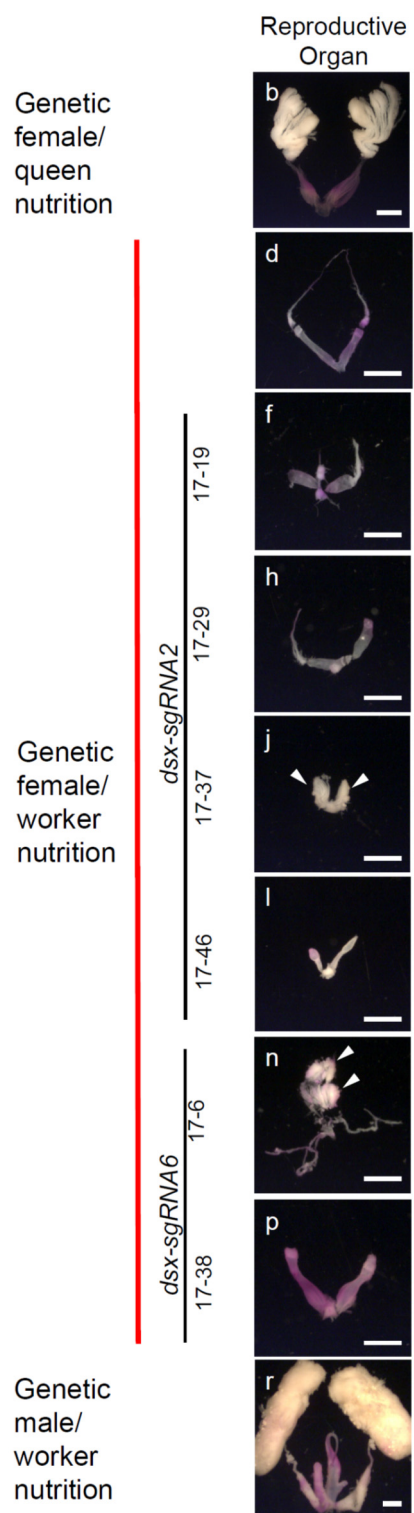

Supplement: S5 Fig — Scale bar, 1 mm. The genetic females were double mutant for dsx and reared on worker nutrition. For further details, see legend of Fig 4 in the main text. (PDF) [file pbio.3000171.s005.pdf]

## Head

## Gonads

Genetic  
female/  
Worker  
nutrition

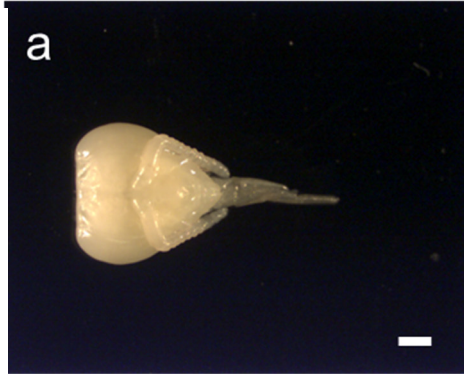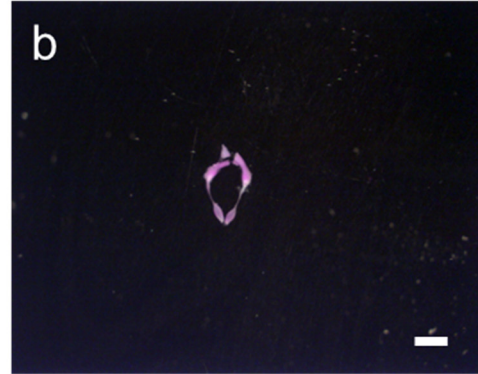

Genetic  
male/  
Worker  
nutrition

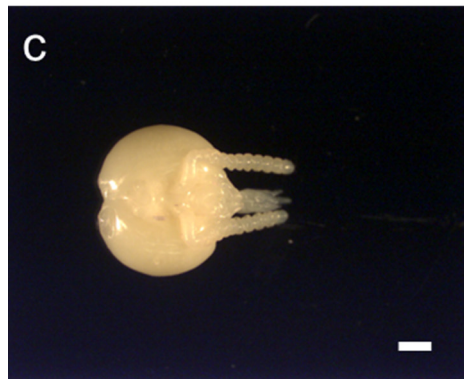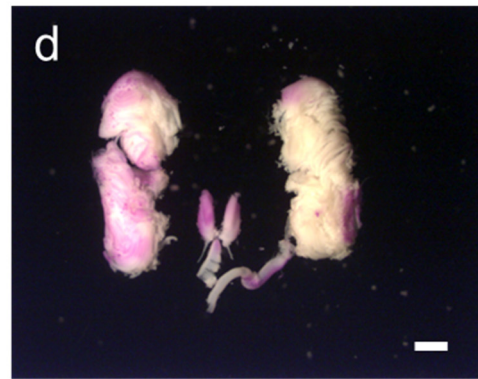

Supplement: S6 Fig — These females have the typical reduced reproductive organ of workers and the fully developed reproductive organs of males. Head and (a) and (c) and reproductive organ (b) and (d). Gonads were stained with aceto-orcein (reddish coloring) to facilitate the dissection process. Scale bar = 1 mm. (PDF) [file pbio.3000171.s006.pdf]
