# Supplementary material for: A genetic switch for worker nutrition-mediated traits in honeybees
Source: PLoS Biol. 2019 Mar 21;17(3):e3000171. doi: 10.1371/journal.pbio.3000171 (PMC6428258; doi:10.1371/journal.pbio.3000171)
Supplement: S2 Fig — (a) Diagrams of the FL analysis for each of the 4 individuals and WT worker bee examples. (b) The nucleotide sequences. We conducted fragment and sequence analysis on amplicons of cDNA to ensure that the many fem-related sequences observed at the fem locus (derived from duplication events) [63] were not amplified. The designated binding sites of the sgRNAs are underlined. Sequence b in larvae #4 resulted from fusion of exon 3 with exon 5. The sequences in larvae #4 resulted from fusion between exon 3 and other fem-related sequences [63]. The WT sequences were obtained from a sample of 5 WT worker larvae (5 clones each). cDNA, complementary DNA; FL, fragment length; WT, wild type. (PDF) [file pbio.3000171.s002.pdf]

a

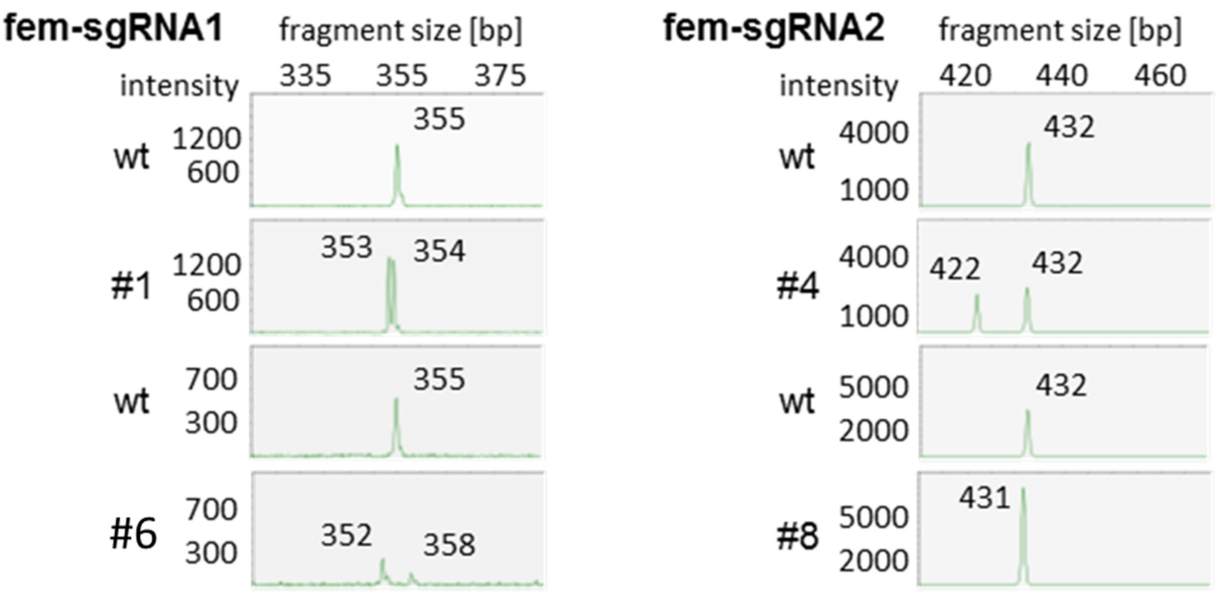

b

Injected sgRNA	Larva No.	Alignment of *fem* sequences
fem-sgRNA1	1	Wildtype AAGATAGCGAAACTGGTCTGCGTTCAAGAACACAAGAAGAACGATT<br>Allele a AAGATAGCGAAACTGGTCTGCGTTCAAGAACACAAGAAGAACGATT<br>Allele b AAGATAGCGAAACTGGTCTGCGTTCAAGAACACAAGAAGAACGATT<br><br>ACGACGTAGACGCGAATGGATGATACAACAAGAACGGGAACGAGAACACGAA<br>ACGACGTAGACG--AATGGATGATACAACAAGAACGGGAACGAGAACACGAA<br>ACGACGTAGACG-GAATGGATGATACAACAAGAACGGGAACGAGAACACGAA
fem-sgRNA1	6	Wildtype TGAAACGGAATACAACAAATCATTCGCATCATGATGAGAGATTTAG<br>Allele a TGAAACGGAATACAACAAATCATTCGCATCATGATGAGAGATTTAG<br>Allele b TGAAACGGAATACAACAAATCATTCGCATCATGATGAGAGATTTAG<br><br>ACAATCACGCAGTGAAGATAGCGAAACTGGTCTGCGTTCAAGAACACAAGAAGAA<br>ACAATCACGCAGTGAAGATAGCGAAACTGGTCTGCGTTCAAGAACACAAGAAGAA<br>ACAATCACGCAGTGAAGATAACAAAATTGATCTGCGTTCAAGAACAAAAGAAGAA<br><br>CGATTACGACGTAGACGCGAA---TGGATGATACAACAAGAACGGGAACGAGAAC<br>CGATTACGACGTAGACGCGAA---TAGAT---ACAACAAGAACGGGAACGAAAAT<br>CGATTACAACATAGACGCGAAGTGTGGTTGATACAACAAGAACGGGAACGAGAAC<br><br>ACGAAAGATTGAAGAAAAAAATGATTTTAGAATACGAATTACGACGTGCTCGTGA<br>ACGAAAGATTGAAGAAAAAAATGATTTTAGAATACGAATTACGACGTGCTCGTGA<br>ACGAAAGATTGAAGAAAAAAATGATTTTAGAATACGAATTACGACGTGCTCGTGA<br><br>GAAAAAATTATCGAAAAGAAGTAAAAGTAGATCCCCAGAAAGCCGAGGTAGAAGT<br>GAAAAAATTATCGAAAAGAAGTAAAAGTAGATCCCCAGAAAGCCGAGGTAGAAGT<br>GAAAAAATTATCGAAAAGAAGTAAAAGTAGATCCCCAGAAAGCCGAGGTAGAAAT<br><br>AATGCATCAAACACGTCTAAAACATTTATATTATCTGAAAAATTAGAATCTTCAG<br>AATGCATCAAACATATCTAAAACATTTATATTATCCGAAAAATTAGAATCTTCAG<br>AATGCATCAAATATATCTAAAACATTTATATTATCCGAAAAATTAGAATCTTCAG<br><br>ATGGTACATCTTTATTTAGAGGACCAGAAGGTACTCAAGTTAGTGCAACAGAACT<br>ATGGTACATCTTTATTTAGAGGACCAGAAGGTACTCAAGTTAGTGCAACAGAACT<br>ATGGTACATTTTTATTTAGAGGACCAGAAGATACTCAAGTTAGTGCAACAGAATT<br><br>ACGAAAAATTAAGGTAGATATTCATAGAGTTTTGCCAGGAAAACCAACAACAACA<br>ACGAAAAATTAAGGTAGATATTCATAGAGTTTTGCTAGGAAAACCAACAACAACA<br>ACAAAAAATTAAAGTAGATATTTATAGGATTTTGCCAGGAAAATCAACAACAACA<br><br>TCTGATGAACTTAAACGGGATATTATCAATCCTGAAGATGTGATGCTCAAAAG<br>TCTGATGAACTTAAACGGGATATTATCAATCCTGAAGATGTGATGCTCAAAAG

		ACTGATGAACTTAAATGAGATATTATCAATCCTGAAGATGTGATGCTCAAAAG
fem-sgRNA2	4	Wildtype sequence for comparison<br>TGAAACGGAATACAACAAATCATTCGCATCATGATGAGAGATTTAGACAATCACG<br>Sequence a (size: -10; 9/38 sequences (24%))<br>TGAAACGGAATACAACAAATCATTCGCATCATGATGAGAGATTTAGACAATCACG<br>Sequence b (size: 0; 24/38 sequences (63%))<br>TGAAACGGAATACAACAAATCATTCGCATCATGATGAGAGATTTAGACAATCACG<br>Sequence c (wildtype; 2/38 sequences (5%))<br>TGAAACGGAATACAACAAATCATTCGCATCATGATGAGAGATTTAGACAATCACG<br>Sequence d (size: 0; 3/38 sequences (8%))<br>TGAAACGGAATACAACAAATCATTCGCATCATGATGAGAGATTTAGACAATCACG<br><br>CAGTGAAGATAGCGAAACTGGTCTGCGTTCAAGAACACAAGAAGAACGATTACGA<br>CAGTGAAGATAGCGAAACTGGTCTGCGTTCAAGAACACAAGAAGAACGATTACGA<br>CAGTGAAGATAGCGAAACTGGTCTGCGTTCAAGAACACAAGAAGAACGATTACGA<br>CAGTGAAGATAGCGAAACTGGTCTGCGTTCAAGAACACAAGAAGAACGATTACGA<br>CAGTGAAGATAGCGAAACTGGTCTGCGTTCAAGAACACAAGAAGAACGATTACGA<br><br>CGTAGACGCGAATGGATGATACAACAAGAACGGGAACGAGAACACGAAAGATTGA<br>CGTAGACGCGAATGGATGATACAACAAGAACGGGAACGAGAACACGAAAGATTGA<br>CGTAGACGCGAATGGATGATACAACAAGAACGGGAACGAGAACACGAAAGATTGA<br>CGTAGACGCGAATGGATGATACAACAAGAACGGGAACGAGAACACGAAAGATTGA<br>CGTAGACGCGAATGGATGATACAACAAGAACGGGAACGAGAACACGAAAGATTGA<br><br>AGAAAAAAATGATTTTAGAATACGAATTACGACGTGCTCGTGAGAAAAAATTATC<br>AGAAAAAAATGATTTTAGAATACGAATTACGACGTGCTCGTGAGAAAAAATTATC<br>AGAAAAAAATGATTTTAGAATACGAATTACGACGTGCTCGTGAGAAAAAATTATC<br>AGAAAAAAATGATTTTAGAATACGAATTACGACGTGCTCGTGAGAAAAAATTATC<br>AGAAAAAAATGATTTTAGAATACGAATTACGACGTGCTCGTGAGAAAAAATTATC<br><br>GAAAAGAAGTAAAAGTAGATCCCCAGAAAGCCGAGGTAGAAGTAATGCATCAAAC<br>GAAAAGAAGTAAAAGTAGATCCCCAGAAAGCCGAGGTAGAAGTAATGCATCAAAC<br>GAAAAGAAGTAAAAGTAGATCCCCAGAAAGCCGAGGTAGAAGTAATGCATCAAAC<br>GAAAAGAAGTAAAAGTAGATCCCCAGAAAGCCGAGGTAGAAGTAATGCATCAAAC<br>GAAAAGAAGTAAAAGTAGATCCCCAGAAAGCCGAGGTAGAAGTAATGCATCAAAC<br><br>ACGTCTAAAACATTTATATTATCTGAAAAATTAGAATCTTCAGATGGTACATCTT<br>ACGTCTAAAACATTTATATTATCTGAAAAATTAGAATCTTCAGATGGTACATCTT<br>ACGTCTAAAACATTTATATTATCTGAAAAATTAGAATCTTCAGATGGTACATCTT<br>ACGTCTAAAACATTTATATTATCTGAAAAATTAGAATCTTCAGATGGTACATCTT<br>ACGTCTAAAACATTTATATTATCTGAAAAATTAGAATCTTCAGATGATATATCTT<br><br>TATTTAGAGGACCAGAAGGTACTCAAGTTAGTGCAACAGAACTACGAAAAATTAA<br>TATTTAGAGGACCAGA----------GTTAGTGCAACAGAACTACGAAAAATTAA<br>TATTTAGAGGACCAGAAGATACTCAAGTTAGTGCAACAGAATTACAAAAAATTAA<br>TATTTAGAGGACCAGAAGGTACTCAAGTTAGTGCAACAGAACTACGAAAAATTAA<br>TATTTAGAGGATTAAAAGGTACTCAAGTTAGTGCAACAGAACTACGAAAAATTAA<br><br>GGTAGATATTCATAGAGTTTTGCCAGGAAAACCAACAACAACATCTGATGAACTT<br>GGTAGATATTCATAGAGTTTTGCCAGGAAAACCAACAACAACATCTGATGAACTT<br>AGTAGATATTTATAGGATTTTGCCAGGAAAATCAACAACAACAACTGATGAACTT<br>GGTAGATATTCATAGAGTTTTGCCAGGAAAGCCAACAACAATATCTGATGAACTT<br>GGTAGATATTCATAGAGTTTTGCTAGGAAAACCAACAACAACATCTGATGAACTT<br><br>AAACGGGATATTATCAATCCTGAAGATGTGATGCTCAAAAG<br>AAACGGGATATTATCAATCCTGAAGATGTGATGCTCAAAAG<br>AAATGAGATATTATCAATCCTGAAGATGTGATGCTCAAAAG<br>AAACGGGATATTATCAATCCTGAAGATGTGATGCTCAAAAG<br>AAACGGGATATTATCAATCCTGAAGATGTGATGCTCAAAAG
fem-sgRNA2	8	Wildtype GGTACATCTTTATTTAGAGGACCAGAAGGTACTCAAGTTAGTGCAA<br>Allele a GGTACATCTTTATTTAGAGGACCAGAAG-TACTCAAGTTAGTGCAA<br>Allele b GGTACATCTTTATTTAGAGGACCAGAAG-TACTCAAGTTAGTGCAA<br><br>CAGAACTACGAAAAATTAAGGTAGATATTCATAGAGTTTTGCCAGGAAAACCAAC<br>CAGAACTACGAAAAATTAAGGTAGATATTCATAGAGTTTTGCCAGGAAAACCAAC<br>CAGAACTACGAAAAATTAAGGTAGATATTCATAGAGTTTTGCCAGGAAAACCAAC
