# Supplementary material for: A genetic switch for worker nutrition-mediated traits in honeybees
Source: PLoS Biol. 2019 Mar 21;17(3):e3000171. doi: 10.1371/journal.pbio.3000171 (PMC6428258; doi:10.1371/journal.pbio.3000171)
Supplement: S3 Fig — The dsx WT nucleotide sequences are represented as a reference sequence. NGS, next-generation sequencing; WT, wild type. (PDF) [file pbio.3000171.s003.pdf]

Injected sgRNA	Larva No.	Alignment of *dsx* sequences
dsx-sgRNA2	17-19	Wildtype CGATGTCTGAATCATCGGCTGGAGATCACCT<br>Allele ....---...............
dsx-sgRNA2	17-29	Wildtype CGATGTCTGAATCATCGGCTGGAGATCACCT<br>Allele a ....----.................<br>Allele b ....---.................
dsx-sgRNA2	17-37	Wildtype CGATGTCTGAATCATCGGCTGGAGATCACCT<br>Allele a ....---...............<br>Allele b ....--...............
dsx-sgRNA2	17-46	Wildtype CGATGTCTGA----------ATCATCGGCTG<br>Allele a ....-------------........<br>Allele b ..........TCATGATCCTGC.........
dsx-sgRNA6	17-6	Wildtype GCATCCTCACACTGCGATGGTCACCCATTTG<br>Allele a ....--...............<br>Allele b ....-................
dsx-sgRNA6	17-38	Wildtype GCATCCTCACACTGCGATGGTCACCCATTTG<br>Allele a ....---................<br>Allele b ....-----..............
