# Supplementary material for: A genetic switch for worker nutrition-mediated traits in honeybees
Source: PLoS Biol. 2019 Mar 21;17(3):e3000171. doi: 10.1371/journal.pbio.3000171 (PMC6428258; doi:10.1371/journal.pbio.3000171)
Supplement: S1 Table — (PDF) [file pbio.3000171.s007.pdf]

		Numbers	Worker phenotypes		
			Head 1) (triangular shaped; upper part straight between compound eyes)	Size of the female reproductive organ 2) (length < 2,5 mm; < 0.7 times the size of the head width)	Ovariole numbers 3) (< 25)
Genetic female	Worker diet in colony	14	14 (100%)	14 (100%)	14 (100%)
	Manually reared on worker nutrition	15	15 (100%)	15 (100%)	15 (100%)

1) Frontal view of head. In contrast to workers, queens have a roundish shaped head; the upper part is curved between compound eyes (see Fig. 4a and b in the main text).

2) Length between the fused left and right part of the reproductive organ to its end in the sagittal plane. The length in queens is > 6 mm and > 1.2 times the size of the head width.

3) Ovariole number in queens is > 100 [11]
