# Supplementary material for: A genetic switch for worker nutrition-mediated traits in honeybees
Source: PLoS Biol. 2019 Mar 21;17(3):e3000171. doi: 10.1371/journal.pbio.3000171 (PMC6428258; doi:10.1371/journal.pbio.3000171)
Supplement: S2 Table — (PDF) [file pbio.3000171.s008.pdf]

	Numbers	Male phenotype	
		Head [1]<br>(round head; complex eyes nearly meet in the upper part)	Size of the male reproductive organ [2]<br>(length > 6 mm;<br>> 1.2 times the width of head)
Male diet in colony	8	8 (100%)	8 (100%)
Manually reared on worker nutrition	20	20 (100%)	20 (100%)

1) Frontal view of head

2) Length between the fused left and right part of the reproductive organ to its end (sagittal plane). The length in workers is < 2.5 mm and < 0.7 times the size of head width.
