# Supplementary material for: A genetic switch for worker nutrition-mediated traits in honeybees
Source: PLoS Biol. 2019 Mar 21;17(3):e3000171. doi: 10.1371/journal.pbio.3000171 (PMC6428258; doi:10.1371/journal.pbio.3000171)
Supplement: S3 Table — Sequences complementary to the designated genomic target site are shown in bold letters. sgRNA, single guide RNA. (PDF) [file pbio.3000171.s009.pdf]

Molecule	Nucleotide sequence
fru-sgRNA1	**GAAUGCACCAGGCAUGUGCG**GUUUUAGAGCUAGAAAUAGCAAGUUAAAAUAAGGCUAG UCCGUUAUCAACUUGAAAAAGUGGCACCGAGUCGGUGCUUUU
fru-sgRNA4	**GCUGGCGGAGGUUGGGCGAC**GUUUUAGAGCUAGAAAUAGCAAGUUAAAAUAAGGCUAG UCCGUUAUCAACUUGAAAAAGUGGCACCGAGUCGGUGCUUUU
fru-sgRNA5	**GCCCGCUGCUGUUCACUCUU**GUUUUAGAGCUAGAAAUAGCAAGUUAAAAUAAGGCUAG UCCGUUAUCAACUUGAAAAAGUGGCACCGAGUCGGUGCUUUU
fem-sgRNA1	**GAUUACGACGUAGACGCGAA**GUUUUAGAGCUAGAAAUAGCAAGUUAAAAUAAGGCUAG UCCGUUAUCAACUUGAAAAAGUGGCACCGAGUCGGUGCUUUU
fem-sgRNA2	**GCACUAACUUGAGUACCUUC**GUUUUAGAGCUAGAAAUAGCAAGUUAAAAUAAGGCUAG UCCGUUAUCAACUUGAAAAAGUGGCACCGAGUCGGUGCUUUU
loc-sgRNA1	**GGCUGGAAUACCGGAAUUCG**GUUUUAGAGCUAGAAAUAGCAAGUUAAAAUAAGGCUAG UCCGUUAUCAACUUGAAAAAGUGGCACCGAGUCGGUGCUUUU
loc-sgRNA2	**GAACGUGGUCUUCACCUUCA**GUUUUAGAGCUAGAAAUAGCAAGUUAAAAUAAGGCUAG UCCGUUAUCAACUUGAAAAAGUGGCACCGAGUCGGUGCUUUU
dsx-sgRNA1	**CTTGCTCGTTTTGTCTCGGC**GUUUUAGAGCUAGAAAUAGCAAGUUAAAAUAAGGCUAG UCCGUUAUCAACUUGAAAAAGUGGCACCGAGUCGGUGCUUUU
dsx-sgRNA2	**CACGTGCTACAGACTTAGTA**GUUUUAGAGCUAGAAAUAGCAAGUUAAAAUAAGGCUAG UCCGUUAUCAACUUGAAAAAGUGGCACCGAGUCGGUGCUUUU
dsx-sgRNA6	**CAACGUAGGAGUGUGACGCU**GUUUUAGAGCUAGAAAUAGCAAGUUAAAAUAAGGCUAG UCCGUUAUCAACUUGAAAAAGUGGCACCGAGUCGGUGCUUUU
