# Supplementary material for: A genetic switch for worker nutrition-mediated traits in honeybees
Source: PLoS Biol. 2019 Mar 21;17(3):e3000171. doi: 10.1371/journal.pbio.3000171 (PMC6428258; doi:10.1371/journal.pbio.3000171)
Supplement: S4 Table — WT, wild type. (PDF) [file pbio.3000171.s010.pdf]

		No. of length-modified sequences [1)]			Sum
		1	2	3	
No. of larvae	Without wt allele (%)	10 (26%)	20 (51%)	1 (3%)	31 (79%)
	With wt allele (%)	8 (21%)	0 (0%)	0 (0%)	8 (21%)

1) Determined by comparing the sequence length of the treated larvae with a sample of 7 non-treated (wild-type, (wt)) larvae.
