# Supplementary material for: A genetic switch for worker nutrition-mediated traits in honeybees
Source: PLoS Biol. 2019 Mar 21;17(3):e3000171. doi: 10.1371/journal.pbio.3000171 (PMC6428258; doi:10.1371/journal.pbio.3000171)
Supplement: S5 Table — At least 10 clones for each larvae were sequenced. These nucleotide changes were consistently not observed in 7 nontreated (WT) larvae. The sequence complementary to the sgRNAs are underlined. sgRNA, single guide RNA; WT, wild type. (PDF) [file pbio.3000171.s011.pdf]

Injected sgRNA	Larva No.	Alignment of the nucleotide sequence at the target sites
fru-sgRNA4	1	Wildtype GAGGGGACGGGTGGAAGCTGGCGGAGGTTGGGCGACGGGGGTGGCG<br>Allele a GAGGGGACGGGTGGAAGCTGGCGGAGGTTGGGC-------------<br>Allele b GAGGGGACGGGTGGAAGCTGGCGGAGG-------------------<br><br>GCCGATTCTCGGTTGGTGGTAGTAGTGGCGGAGGCTAAAGGGGAAAGGGGGGTGG<br>--CGATTCTCGGTTGGTGGTAGTAGTGGCGGAGGCTAAAGGGGAAAGGGGGGTGG<br>----------------------------------CTAAAGGGGAAAGGGGGGTGG
fru-sgRNA4	2	Wildtype GGAGGGGACGGGTGGAAGCTGGCGGAGGTTGGGCGACGGGGGTGGC<br>Allele a GGAGGGGACGGGTGGAAGCTG--------------ACGGGGGTGGC<br>Allele b GGAGGGGACGG-----------------------------------<br><br>GGCCGATTCTCGGTTGGTGGTAGTAGTGGCGGAGGCTAAAGGGGAAAGGGGGGTG<br>GGCCGATTCTCGGTTGGTGGTAGTAGTGGCGGAGGCTAAAGGGGAAAGGGGGGTG<br>---<br><br>GCGGGAGTGGCGGCGGCGGCGGCGGCGGCGGCGGCGGCGAACATAAAATCCCTCG<br>GCGGGAGTGGTGGCGGCGGCGGCGGCGGCGGCGGCGGCGAACATAAAATCCCTCG<br>-------------------------------------CGAACATAAAATCCCTCG<br><br>CACAT<br>CACAT<br>CACAT
fru-sgRNA4	3	Wildtype AGTGGCGGGGGAGGAGGGTCGGAGGGGACGGGTGGAAGCTGGCGGA<br>Allele a AGTGGCGGGGGAGGAGGGTCGGAGGGGACGGGTGGAAGCTGGCGGA<br>Allele b AGTGGCGGGGGAGGAGGGTCGGAGGGGACGGGTGGAAGCTGGCGGA<br><br>GGTTGGGC-GACGGGGGTGGCGGCCGATTCTCGGTTGGTGGTAGTAGTGGCGGAG<br>GGTTGGCCCGACGGGGGTGGCGGCCGATTCTCGGTTGGTGGTAGTAGTGGCGGAG<br>GGTTG-----ACGGGGGTGGCGGCCGATTCTCGGTTGGTGGTAGTAGTGGCGGAG
fru-sgRNA5	2	Wildtype GGCTTCAACGCGGCTCGGTTGGGTGGTGGCCCGCTGCTGTTCACTC<br>Allele a GGCTTCAACGCGGCTCGGTTGGGTGGTGACCCGCTGCTATTCCCT-<br>Allele b GGCTTCAACGCGGCTCGGTTGGGTGGTGGCCCGCTGCTGTTCACT-<br><br>TTTGGAGAGGAAAGGGTTGCGCGAGGAGCGACGGGGACAGGGTGGGAAAAAGAGA<br>-TTGGAGAGGAAAGGGTTGCGCGAGGAGCGACGGGGACAGGGTGGGAAAAAGAGA<br>-TTGGAGAGGAAAGGGTTGCGCGAGGAGCGACGGGGACAGGGTGGGAAAAAGAGA
fru-sgRNA5	4	Wildtype GTTGGGTGGTGGCCCGCTGCTGTTCACTCTTTGGAGAGGAAAGGGT<br>Allele a GTTGGGTGGTGGCCCGCTGCTGTTC---CTTTGGAGAGGAAAGGGT<br>Allele b GTTGGGTGGTGGCCCGCTGCTGTTCA--CTTTGGAGAGGAAAGGGT<br><br>TGCGCGAGGAGCGACGGGGACAGGGTGGGAAAAAGAGAGGGATGCGTGAAGGAGG<br>TGCGCGAGGAGCGACGGGGACAGGGTGGGAAAAAGAGAGGGATGCGTGAAGGAGG<br>TGCGCGAGGAGCGACGGGGACAGGGTGGGAAAAAGAGAGGGATGCGTGAAGGAGG<br><br>AAGGGTGAAGAACGAGGGAAAGGAGGAAGAGGAGGAGGACGGAGGAGAAGGAGGA<br>AAGGGTGAAGAACGAGGGAAAGGAGGAAGAGGAGGAGGACGGAGGAGAAGGAGGA<br>AAGGGTGAAGAACGAGGGAAAGGAGGAAGAGGAGGAGGACGGAGGAG---GAGGA<br><br>GGTGGGGGAGAGTGG<br>GGTGGGGGAGAGTGG<br>GGTGGGGGAGAGTGG
fru-sgRNA5	7	Wiltdype GGCTTCAACGCGGCTCGGTTGGGTGGTGGCCCGCTGCTGTTCACTC<br>Allele a GGCTTCAACGCGGCTCGGTTGGGTGGTGGCCCGCTGCTGTTCACT-<br>Allele b GGCTTCAACGCGGCTCGGTTGGGTGGTGGCCCGCTGCTGTT-----<br><br>TTTGGAGAGGAAAGGGTTGCGCGAGGAGCGACGGGGACAGGGTGGGAAAAAGAGA<br>--TGGAGAGGAAAGGGTTGCGCGAGGAGCGACGGGGACAGGGTGGGAAAAAGAGA<br>--TGGAGAGGAAAGGGTTGCGCGAGGAGCGACGGGGACAGGGTGGGAAAAAGAGA
dsx-sgRNA1	12	Wildtype TGTGAATCGAGGTTACCTATGTATCGCGAAGAGAACGAGCAAAACA<br>Allele a TGTGAATCGAGGTTACCTATGTATCGCGAAGAGAACGAGCAAAACA<br>Allele b TGTGAATCGAGGTTACCTATGTATCGCGAAGAGAACGAGCAAAACA<br><br><br>GAGCCGCGGACTTGGCTCCCCAACAACCGAGTGGTGCAAACACGTTCGAGCGTTT

		GAGCCGCGGACTTGGCTCCCCAACAACCGAGTGGTGCAAACACGTTCGAGCGTTT<br>GAGC---GGACTTGGCTCCCCAACAACCGAGTGGTGCAAACACGTTCGAGCGTTT
dsx-sgRNA1	16	Wildtype TGTGAATCGAGGTTACCTATGTATCGCGAAGAGAACGAGCAAAACA<br>Allele a TGTGAATCGAGGTTACCTATGTATCGCGAAGAGAACGAGCAAAACA<br>Allele b TGTGAATCGAGGTTACCTATGTATCGCGAAGAGAACGAGCAAAACA<br><br>GAGCCGCGGACTTGGCTCCCCAACAACCGAGTGGTGCAAACACGTTCGAGCGTTT<br>GAGCCGCGGACTTGGCTCCCCAACAACCGAGTGGTGCAAACACGTTCGAGCGTTT<br>GAGT---GGACTTGGCTCCCCAACAACCGAGTGGTGCAAACACGTTCGAGCGTTT
dsx-sgRNA2	9	Wildtype TACTCCAAAGCCGCGTGCACGGAATTGTGCACGATGTCTGAAT---<br>Allele a TACTCCAAAGCCGCGTGCACGGAATTGTGCACGATGTCTGATCGGC<br>Allele b TACTCCAAAGCCGCGTGCACGGAATTGTGCACGATGTCTGAAGATC<br><br>----------CATCGGCTGGAGATCACCTTAAAATCGCACAAGAGGTACTGTGTA<br>TGAATCGGCACATCGGCTGGAGATCACCTTAAAATCGCACAAGAGGTACTGTGTA<br>---------ACATCGGCTGGAGATCACCTTAAAATCGCACAAGAGGTACTGTGTA
dsx-sgRNA2	11	Wildtype TACTCCAAAGCCGCGTGCACGGAATTGTGCACGATGTCTGAATCAT<br>Allele a TACTCCAAAGCCGCGTGCACGGAATTGTGCACGATGTCTGAAGCTG<br>Allele b TACTCCAAAGCCGCGTGCACGGAATTGTGCACGATGTCTGAAGCTG<br><br>CGGCTGGAGATCACCTTAAAATCGCACAAGAGGTACTGCAAGTACCGTACTTGTA<br>TCGT--GAGATCACCTTAAAATCGCACAAGAGGTACTGCAAGTACCGTACTTGTA<br>TCGT--GAGATCACCTTAAAATCGCACAAGAGGTACTGCAAGTACCGTACTTGTA
dsx-sgRNA2	68	Wildtype TACTCCAAAGCCGCGTGCACGGAATTGTGCACGATGTCTGAATCAT<br>Allele a TACTCCAAAGCCGCGTGCACGGAATTGTGCACGATGTCTGAATCAT<br>Allele b TACTCCAAAGCCGCGTGCACGGAATTGTGCACGATGTCTGAATCGT<br><br>CGGCTGGAGATCACCTTAAAATCGCACAAGAGGTACTGCAAGTACCGTACTTGTA<br>CGGCTGGAGATCACCTTAAAATCGCACAAGAGGTACTGCAAGTACCGTACTTGTA<br>--GCTGGAGATCACCTTAAAATCGCACAAGAGGTACTGCAAGTACCGTACTTGTA
loc552773-sgRNA1	3	Wildtype CGATCGATCAGCTTCGTGACAAATTATCGGCTGGAATACCGGAAT-<br>Allele a CGATCGATCAGCTTCGTGACAAATTATCGGCTGGAATACCGGAAGG<br>Allele b CGATCGATCAGCTTCGTGACAAATTATCGGCTGGAATACCGGCTGG<br><br>----------TCGAGGCGCCACCCATCGAGCCCCTATACCTCAAGCAAATTCGCC<br>CGCCACCCATTCGAGGCGCCACCCATCGAGCCCCTATACCTCAAGCAAATTCGCC<br>AATA------TCGAGGCGCCACCCATCGAGCCCCTATACCTCAAGCAAATTCGCC
loc552773-sgRNA1	6	Wildtype CGATCGATCAGCTTCGTGACAAATTATCGGCTGGAATACCGGAAT-<br>Allele a CGATCGATCAGCTTCGTGACAAATTATCGGCTGGAATACCGGAAGG<br>Allele b CGATCGATCAGCTTCGTGACAAATTATCGGCTGGAATACCGGAAGG<br><br>---TCGAGGCGCCACCCATCGAGCCCCTATACCTCAAGCAAATTCGCCTGTCCAG<br>TATTCGAGGCGCCACCCATCGAGCCCCTATACCTCAAGCAAATTCGCCTGTCCAG<br>TATTCGAGGCGCCACCCATCGAGCCCCTATACCTCAAGCAAATTCGCCTGTCCAG
loc552773-sgRNA1	7	Wildtype CGATCGATCAGCTTCGTGACAAATTATCGGCTGGAATACCGGAAT-<br>Allele a CGATCGATCAGCTTCGTGACAAATTATCGGCTGGAATACCGG----<br>Allele b CGATCGATCAGCTTCGTGACAAATTATCGGCTGGAATACCGGAAGG<br><br>---TCGAGGCGCCACCCATCGAGCCCCTATACCTCAAGCAAATTCGCCTGTCCAG<br>-----AAGGCGCCACCCATCGAGCCCCTATACCTCAAGCAAATTCGCCTGTCCAG<br>TATTCGAGGCGCCACCCATCGAGCCCCTATACCTCAAGCAAATTCGCCTGTCCAG
loc552773-sgRNA2	9	Wildtype GTTCGAGATCTCAAAGCGGATGTCGAGAACGTGGTCTTCACCTTCA<br>Allele a GTTCGAGATCTCAAAGCGGATGTCGAGAACGTGGTCTTCACTTTCA<br>Allele b GTTCGAGATCTCAAAGCGGATGTCGAGAACGTGGTCTTCACC-TCA<br><br>AGGTGAACTTTGAGAAACTCCATTTCCAAGGGAAGTATCAGATCGACGCGAGGGT<br>AGGTCAACTTTGAGAAACTCCATTTCCAAGGGAAGTATCAGATCGACGCGAGGGT<br>AGGTGAACTTTGAGAAACTCCATTTCCAAGGGAAGTATCAGATCGACGCGAGGGT
