# Supplementary material for: A genetic switch for worker nutrition-mediated traits in honeybees
Source: PLoS Biol. 2019 Mar 21;17(3):e3000171. doi: 10.1371/journal.pbio.3000171 (PMC6428258; doi:10.1371/journal.pbio.3000171)
Supplement: S6 Table — CRISPR/Cas9, clustered regularly interspaced short palindromic repeats/CRISPR-associated protein 9. (PDF) [file pbio.3000171.s012.pdf]

Indels (bp)	#	Relative frequency
> -20	2	8%
-20 to -11	3	12%
-10 to -6	2	8%
-5 to -1	11	44%
+1 to +5	5	20%
+6 to +10	0	0%
+11 to +20	2	8%
> +20	0	0%
