# Supplementary material for: A genetic switch for worker nutrition-mediated traits in honeybees
Source: PLoS Biol. 2019 Mar 21;17(3):e3000171. doi: 10.1371/journal.pbio.3000171 (PMC6428258; doi:10.1371/journal.pbio.3000171)
Supplement: S7 Table — (PDF) [file pbio.3000171.s013.pdf]

Injected sgRNA	Larva No.	Alignment of the hypervariable region of the *csd* alleles
fem-sgRNA1	1	*csd* allele 1 GAACCTAAAATAATTTCATCTTTATCGAACAATTACAATTAT<br>*csd* allele 2 GAACCTAAAATAATTTCATCTTTATCGAGCAATTACAATTCT<br><br>AATAATAATAATTATAATAATTATAATAATTATAATAATTATAATAATAATTATA<br>AACAATTATAATAATTATAGTACTAATTAT-------------------------<br><br>ATAATAATTATAATAAAAAATTA------TATTACAATATTAATTATATTGAACA<br>--------------AAACAATTACAATATTGTTACAATATTAATTATATTGAACA<br><br>AATTCCTGTTCCTGTT<br>AATTCCTATTCCTGTT
fem-sgRNA1	6	*csd* allele 3 GAACCTAAAATAATTTCATCTTTATCGAATAAGACAATACAT<br>*csd* allele 4 GAACCTAAAATAACTTCATCTTTATCGAACAATTACAATTCT<br><br>AATAATAATAATTAT---------------------------AAAAAATTATATT<br>AATAATTATAATAATTATAATAAATATAATTATAATAATTCTAAAAAATTATATT<br><br>ACAATATTAATTATATTGAACAAATTCCTATTCCTGTT<br>ACAATATTAATTATATTGAACAAATTCCTGTTCCTATT
fem-sgRNA2	4	*csd* allele 5 GAACCTAAAATAATTTCATCTTTATCGAACAAGACAATACAT<br>*csd* allele 6 GAACCTAAAATAATTTCATCTTTATCGAACAATTACAATTAC<br><br>AATAATAATAAATATAATTATAATAATAATTATAATAATAATTGTAAAAAATTAT<br>AACAATTATAATAATAATTAT------------------------AAACCATTAT<br><br>ATTACAATATTAATTATATTGAACAAATTCCTATTCCTGTT<br>ATTACAATATTAATTATATTGAACAAATTCCTGTTCCTGTT
fem-sgRNA2	8	*csd* allele 8 GAACCTAAAATAATTTCATCTTTATCGAATAATACAATACAT<br>*csd* allele 9 GAACCTAAAATAATTTCATCTTTATCGAACAAGACAATACAT<br><br>AATAATAATTATAAATATAATTATAATAATAATTATAATAA---TTATAAAAAAT<br>AATAATAAT---AAATATAATTATAATAATAATTATAATAATAATTGTAAAAAAT<br><br>TATATTACAATATTAATTATATTGAACAAATTCCCGTTCCTGTT<br>TATATTACAATATTAATTATATTGAACAAATTCCTATTCCTGTT
