# Supplementary material for: A genetic switch for worker nutrition-mediated traits in honeybees
Source: PLoS Biol. 2019 Mar 21;17(3):e3000171. doi: 10.1371/journal.pbio.3000171 (PMC6428258; doi:10.1371/journal.pbio.3000171)
Supplement: S8 Table — (PDF) [file pbio.3000171.s014.pdf]

Treatment	Number	Number of bees with large gonads (male type) and double nonsense mutations
fem-sgRNA<br>(sgRNA1/sgRNA2)[1)]	27<br>(11/16)	4 (15%)<br>(2 /2)
Untreated	38	0 (0%)

1) 400 pg *Cas9 mRNA* together with 5.5 pg *fem-sgRNA1* or 14.6 pg *fem-sgRNA2* were injected per embryo.
