# Supplementary material for: A genetic switch for worker nutrition-mediated traits in honeybees
Source: PLoS Biol. 2019 Mar 21;17(3):e3000171. doi: 10.1371/journal.pbio.3000171 (PMC6428258; doi:10.1371/journal.pbio.3000171)
Supplement: S9 Table — (PDF) [file pbio.3000171.s015.pdf]

		Numbers	Length at larval stage 5	
			> 2.5 mm (Testis)	< 1.5 mm (Ovary)
Genetic female/ manually reared on worker nutrition	double missense mutation in *fem*	4	4 (100%)	0
	Wildtype	38	0	38 (100%)
