# Supplementary material for: A genetic switch for worker nutrition-mediated traits in honeybees
Source: PLoS Biol. 2019 Mar 21;17(3):e3000171. doi: 10.1371/journal.pbio.3000171 (PMC6428258; doi:10.1371/journal.pbio.3000171)
Supplement: S10 Table — (PDF) [file pbio.3000171.s016.pdf]

Treatment	Experiment	Numbers of bees at larval stage 1	Numbers of bees at stage of phenotyping[1)]	Bees with worker head [2)]	Bees with intersex reproductive organ	% of intersexes with *dsx* double mutations
dsx-sgRNA2	1	125	41	41 (100%)	4 (10%)	100%
	2	362	11	11 (100%)	5 (45%)	100%
dsx-sgRNA6		39	11	11 (100%)	2 (18%)	100%
No treatment		82	34	34 (100%)	0%[3)]	0%

1) Genetic mosaics were excluded

2) Frontal view: triangular shaped; upper part straight between compound eyes

3) 17 out of 34 were dissected
